# The Impact of a Community Awareness Strategy on Caregiver Treatment Seeking Behaviour and Use of Artemether-Lumefantrine for Febrile Children in Rural Kenya

**DOI:** 10.1371/journal.pone.0130305

**Published:** 2015-07-02

**Authors:** Beatrice Wasunna, Emelda A. Okiro, Jayne Webster, Jim Todd, Robert W. Snow, Caroline Jones

**Affiliations:** 1 Eastern and Southern Africa Centre of International Parasite Control (ESACIPAC), Kenya Medical Research Institute, P.O. Box 54840–00200, Nairobi, Kenya; 2 Department of Public Health Research, Kenya Medical Research Institute/Wellcome Trust Research Programme, Centre for Geographic Medicine Research-Coast (CGMRC), P.O. Box 43640–00100 GPO, Nairobi, Kenya; 3 Centre for Tropical Medicine, Nuffield Department of Clinical Medicine, University of Oxford, CCVTM, Oxford, United Kingdom; 4 Disease Control Department, London School of Hygiene and Tropical Medicine (LSHTM), London, Keppel Street, WCIE 7HT, London, United Kingdom; 5 Department of Population Health, London School of Hygiene and Tropical Medicine, London, United Kingdom, Keppel Street, WCIE 7HT, London, United Kingdom; 6 Health Systems and Social Science Research, Kenya Medical Research Institute/Wellcome Trust Research Programme, Centre for Geographic Medicine Research-Coast (CGMR-C), P.O. Box 230, Kilifi, Kenya; University of Washington, UNITED STATES

## Abstract

**Background:**

Access to prompt and effective treatment is the cornerstone for malaria control. Population Services International in collaboration with the Ministry of Health launched a malaria behaviour change communication intervention in Nyanza province, Kenya. The initiative aimed to improve: symptom recognition and prompt access to government health facilities for febrile children; effective treatment with the recommended first-line drug artemether-lumefantrine (AL) in public health facilities and adherence to the AL regimen.

**Methods:**

Pre- and post-intervention cross-sectional household surveys were used to evaluate the impact of the intervention on prompt and correct use of AL for febrile children below five years of age. The primary outcome was the proportion of children below five years of age with fever in the last 14 days accessing AL within 48 hours of fever onset.

**Results:**

There was an increase from 62.8% pre-intervention to 79.4% post-intervention (95% CI: 11.1, 22.1) in caregivers who reported seeking formal treatment promptly (on the same day, or next day) for their febrile children. However, there was a decrease in the use of government health facilities in the post-intervention period. There was a small increase in the proportion of children accessing AL within 48 hours of fever onset [18.4% vs 23.5% (0.1–10.0)].

**Conclusion:**

The findings of this evaluation demonstrate that interventions that target only one sector may have a limited impact on improvements in prompt and effective treatment where multiple sources of treatments are sought for febrile illness. Additionally, the context in which an intervention is implemented is likely to influence the process and outcomes.

## Introduction

In September, 2008, the Roll Back Malaria (RBM) partnership launched the Global Malaria Action Plan (GMAP) to provide a framework for action around which stakeholders in malaria endemic nations could coordinate their malaria control efforts [1]. As part of this strategy pre-elimination countries were expected to make sure that at least 80% of febrile children in malaria endemic regions accessed effective antimalarial treatment early in their illness by 2015 [1]. By 2006 every country in sub-Saharan Africa had abandoned failing monotherapies in favor of artemisinin-based combination therapy (ACT) as recommended first-line treatment for uncomplicated malaria [2], but, despite these policy changes, recent data suggest that there was a large variation in the proportion of children under five with a fever treated with an antimalarial who received an ACT ranging from under 5% in Chad to over 90% in Rwanda [3].

In Kenya, the first-line antimalarial drug policy was changed officially in 2004 from sulphadoxine-pyrimethamine (SP) to artmether-lumefantrine (AL) but it wasn’t until late 2006 that AL was procured and distributed to all levels of the government and mission health sectors alongside revised standard treatment guidelines and in-service training for health care providers [4]. Despite a few pilot interventions targeting retail-sector provision, AL remained a prescription-only medicine and largely confined to formal public health services across Kenya [5] through to the end of 2010. Cross-sectional health facility surveys undertaken in Kenya, four to six months after AL policy roll out, reported that only 28% of children meeting the national guidelines definition of uncomplicated malaria were prescribed AL, despite AL being in stock on the day of the survey [6]. Even fewer children were receiving the effective antimalarial early in their illness with studies undertaken at various sites in Kenya between 2006 and 2007, reporting that only 11% of febrile children below 5 years received AL within 48 hours of fever onset [7].

In an attempt to address the large gap between the GMAP targets and the proportion of febrile children under five accessing AL from public sector services early in their illness in Kenya, Population Services International (PSI) in collaboration with the Ministry of Public Health and Sanitation (MoPHS) developed and implemented an intensive malaria case management behavioral change initiative. The initiative was undertaken during the previous malaria treatment policy recommendations of presumptive treatment of all fevers in malaria endemic regions of Kenya. This initiative began in February 2009 with financial support from the Pfizer Mobilize against Malaria program [8] and identified two principal target populations: prescribers of anti-malarials operating from government health facilities and caretakers of young children residing in the community. The impact of the intervention targeting prescribers (enhanced health worker training) has been reported elsewhere [9]. In this paper we focus on the impact of the second aspect of the intervention, a community based behaviour change communication campaign designed to improve the numbers of febrile children attending public health facilities promptly and increase the proportion completing the AL treatment regime. The core theme of the behavioural change initiative was prompt action for all fevers in children branded under the phrase *haraka upesi* (English = prompt action). A professional advertising agency based in Nairobi was employed to work on communication messages, channels and materials following a communications brief provided by PSI that sought to reinforce three key behaviours: 1) the importance of prompt symptom recognition specifically, getting febrile children into clinics promptly, 2) effective treatment with the recommended first-line dug (AL) at a public health facility and 3) adherence to the AL regimen. The channels of communication included mass media (including radio), posters, distribution of messaging materials, message dissemination through road-shows and established community-based organisations. Here we report on the evaluation of the impact of the intervention on the appropriate treatment (time/drug/dose/duration) for children under five who had suffered from a fever in the past two weeks. Community level intervention activities were undertaken between November 2009 and September 2010. Radio messaging was undertaken between October 2009 and September 2010.

## Methods

### Study district

Bondo district was selected as the evaluation district from the twelve districts located in Nyanza Province. Bondo is on the shores of Lake Victoria with intense, perennial malaria transmission with a predicted 2009 parasite prevalence in children in excess of 40% [10], where hospital admissions have not declined since 1999 [11], where previous studies have suggested that access to prompt treatment with AL was poor following the change in drug policy [7] and performance of health workers in the government sector sub-optimal with respect to managing febrile children [6,12]. The district has four main administrative divisions namely: Bondo, Usigu, Madiany and Rarieda. Forty one government and mission health facilities serve the district population of over 280,000 inhabitants and are staffed by over 100 health workers.

### Study design

The study was designed as a pre-post intervention cluster sample survey [13].

A GIS platform was developed including enumeration areas (EA) and associated population data was created based on EAs used during the 1999 national census [14], roads, rivers, schools, health facilities, market centres, administration offices and major landmarks created from national digital data sources and updated through detailed mapping in July 2010. All data were stored in ArcView GIS 3.2 (ESRI Inc., New York, USA) for survey work and estimation of travel distances to health facilities, market centre’s and proximity to posters and CBO small group sessions.

**Fig 1:**
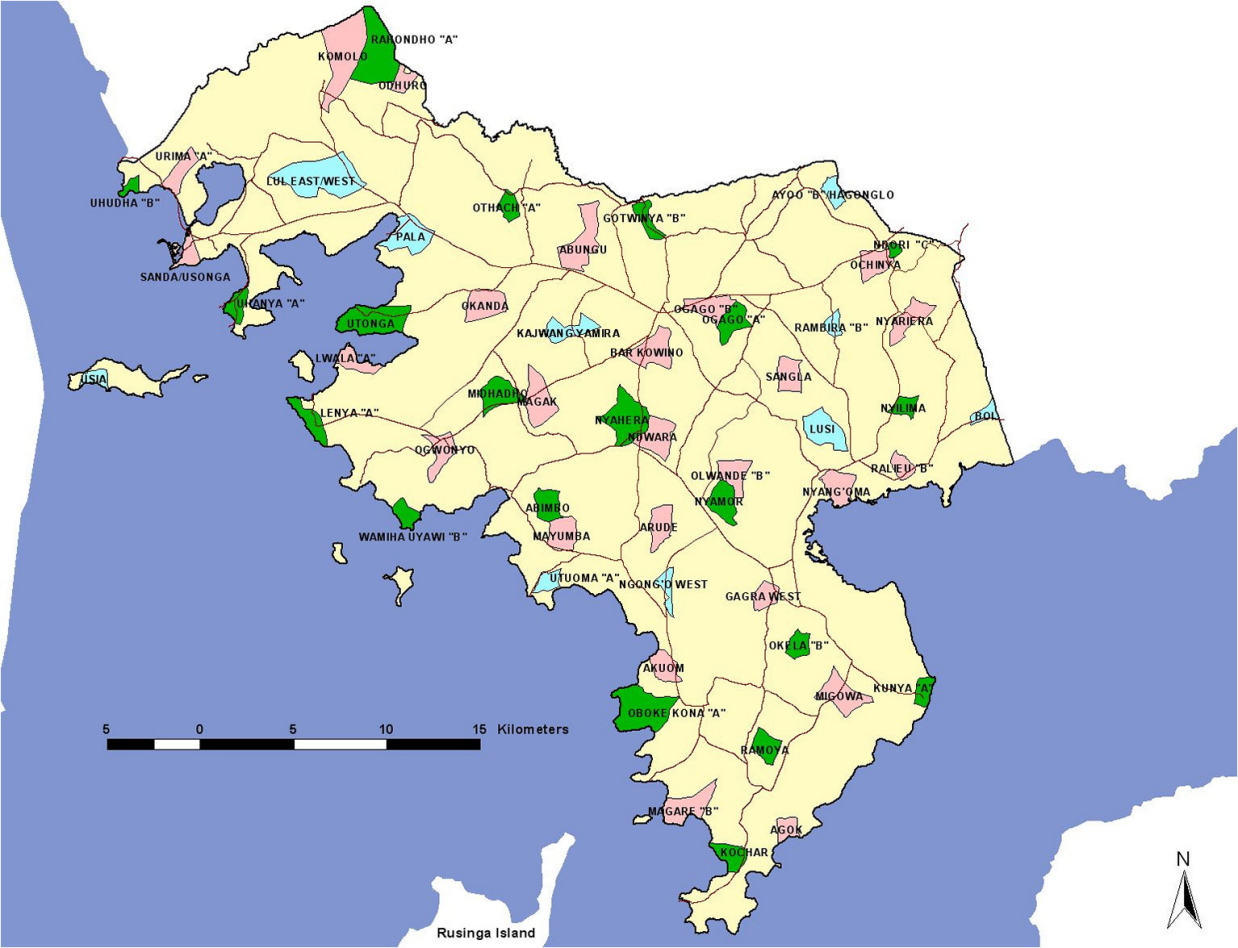
The distribution of randomly selected pre- (Green) and Post-intervention (Pink) enumeration area clusters for the household surveys. Blue clusters indicate where ten clusters were randomly selected in both pre- and post-intervention selection

Sample size estimates for each of the household surveys were calculated for the proportion of children who access effective malaria treatment within 48 hours of fever [15]. We assumed an initial proportion of 11% of febrile children access AL within 48 hours on onset of symptoms (primary outcome) as described in 2007 in Bondo district [7] with a 4% minimum point increase post-intervention. Included in the sample size estimation was an estimate of the proportion of children aged less than five years, a 10% non-response rate and a design effect of 2 to adjust for clustering. The anticipated sample size was therefore computed as 600 households located in 30 EA clusters. Two samples were selected, first for the pre-intervention survey and separately for the post-intervention survey. The second sample selection was increased to accommodate an unexpectedly higher AL use at baseline and thus included 700 households located in 35 clusters. Sample EAs were selected using the spatial randomization function in ArcView 3.2 to ensure all areas across the district were equally represented. The distribution of survey clusters is shown in Fig 1. Within each cluster, household were randomly selected using the World Health Organization Expanded Programme of Immunization random walk [16,17].

### Household surveys

Evaluation of community level intervention activities was undertaken through two cross-sectional household surveys. Pre-intervention (baseline) household surveys were undertaken between June and July 2009 and post-intervention surveys undertaken between July and August 2010. Post-intervention surveys were delayed due to inadequate AL stocks in public health facilities in Bondo between May and July 2010. All mothers or caretakers of children aged under-five years were asked whether their child had experienced a fever in the last 14 days and whether the child was febrile on the day of the survey. For each fever the sources, types and timing of treatment actions were documented. A photo-illustrated guide was used to assist recognition of proprietary forms of anti-malarials likely to be purchased from the retail sector and those available in the public sector. Questions were also asked on numbers of tablets taken and mothers were asked to show packaging if available to confirm completed doses. Finally mother’s and caretakers were interviewed about their knowledge of malaria, AL and sources of treatment and whether they had been exposed to any public messages on prompt fever treatment and the source of these messages. A second photo-illustrated guide of all PSI community intervention materials was developed to enable mothers identify IEC materials distributed as part of the intervention. The primary outcome was *‘the proportion of children under five years of age with fever in the last 14 days accessing AL within 48 hours of fever onset’*.

**Adherence** was examined among all fevers where treatment started four or more days preceding the survey day. A participant was defined as adherent if the caregivers presented an empty AL blister pack and/or stated that all medicines had been administered. Non-adherents were those who had leftover tablet (s) in a blister pack where treatment was started four days and/or reported to have not completed the dose that they had been given. Caregivers who used AL already existing in the household were excluded from the adherence analysis.

To establish whether caregivers adhered to AL treatment, questions were asked in the household surveys on the total number of tablets given, those given in a single dose, number of times per day a drug was given, number of days and whether all tablets were given. The number of left over tablets was assessed by requesting to see incomplete AL blister packs (blister packs with left over tablets) and, if not available, by calculating the total number of tablets administered against the total number of tablets received.

Data were captured directly on data screens designed using Pendragon version 5.1 (Pendragon Software Corporation, Libertyville, Illinois) with internal consistency checks, using personal digital assistants (model-HP Ipaq 114 classic handheld, Hewlett-Packard Company, Palo Alto, California) and downloaded into Microsoft Office Excel (2007) comma separated value (CSV) files.

### Assessment of AL availability at government and mission health facilities

Data on drug availability was necessary to interpret the results from the pre- and post-intervention community surveys. Inadequate AL supply at the point of care will inevitably result in severely compromised malaria case management. Telephone interviews and physical audits were undertaken with health facility in-charges in the universe of 36 government and five mission health facilities between January 2009 and June 2010 to assess AL availability.

### Analysis

All analyses were performed using STATA, version 11 (StataCorp, College Station, Texas). A household wealth index was constructed from household characteristics and asset data using principal component analysis (PCA) [18,19]. Weights were derived from the first principal component and used to construct the wealth index that classified households into wealth quintiles, based on whether the household had electricity, radio, bicycle, mobile phone, household head occupation status and level of education, household ownership of cows, main source of drinking water, type of toilet facility, source of fuel for household cooking, household wall construction material, and household floor material.

A descriptive analysis of fever treatment actions was first undertaken to obtain all frequency distributions of relevant variables/indicators for pre-and post-intervention surveys. The analysis accounted for the survey design by adjusting for cluster selection using *svy* command in STATA. The survey design was self-weighting. The sample size and initial hypothesis was based on the proportion of children with fever accessing AL within 48 hours, but the analysis also assessed many different outcomes to assess the impact of the sensitization program. Differences in proportions were compared for significance using the chi-squared test, notably: 1) changes in fever treatment seeking behaviour through a pre- versus post-intervention comparison; 2) access to antimalarial medicines by socio-economic quintile pre and post-intervention; 3) changes in the proportions of febrile children accessing AL at any time within 14 days, and a subset within 48 hours of fever onset pre- and post-intervention; and 4) the changes in the proportions of febrile children accessing AL from public sector facilities within 48 (same day/next day) of fever onset pre- and post-intervention. Logistic regression was used to determine factors associated with prompt (within 48 hours) treatment of fever with AL among children < 5 years of age in each of the surveys. The factors assessed included age of child, child’s gender, caregiver’s age, caregivers’ level of education, number of children below five years of age in the household, socio-economic status and distance to the nearest public health facility. Associations were assessed using adjusted Wald test on the three outcomes. Variables were screened using univariable analysis and those that gave a p value ≤0.05 were considered potential predictors of the treatment outcome. Univariate analysis of factors was performed using logistic regression to take account of clustering. To decide which predictive factors to include in the regression models the likelihood ratio test was used, and assessment of confounding and effect modification was considered only in factors that were associated with the outcome (prompt treatment of fever). The test showed strong evidence (p<0.001) of variability between clusters and between caregivers. Univariable and multivariable logistic regression analysis with the child as the unit of analysis was undertaken using the random intercept model (xtmelogit command) in STATA accounting for clustering at EA and caregiver levels.

### Ethical Approval

Ethical approval for this study was obtained from the KEMRI National Ethical Review Committee (KEMRI SSC number: 1375) and the London School of Hygiene and Tropical Medicine Ethics Committee (Ethical approval number: 5313).

## Results

### Children with fever

Of the 958 children under five years of age sampled in 2009, 506 (52.8%) were reported to have experienced a fever event in the last 14 days. This was a higher proportion than in 2010 when of the 1,123 children under five, 515 (45.9%) were reported to have had fever in the last 14 days (p = 0.006) (Table 1). Of the children with reported fever, 81.8% and 73.2% in 2009 and 2010 respectively were not febrile on the day of the survey.

**Table 1:** Characteristics of surveyed communities, households, children and caregivers pre- and post-intervention. Proportions are cluster adjusted.

	Pre-intervention (June-July 2009)	Post-intervention (July-August 2010)
**Number of clusters**	30	35
Number of households	600	700
Number of children under five	958	1, 123
Number (%) children who were female	483 (50.4%)	558 (49.7%)
Number of children aged <1 year	204 (21.3%)	232 (20.7%)
Number of children aged 1 year	208 (21.7%)	202 (18.0)
Number of children aged ≥ 2 years	546 (57.0%)	689 (61.3)
Number of households with more than one child < 5 years	293 (48.8)	355 (50.7)
Mean Euclidian distance (km) of household to nearest public health facility (+/- SD)	2.6km (1.3)	2.3km (1.2)
**Key household variables used for wealth assets**		
Household head employed in wage economy	99 (16.5%)	88 (12.6%)
Household owner occupied	486 (81.0%)	629 (89.9%)
Source of drinking water—piped water	123 (20.5%)	138 (19.7%)
Source of drinking water—lake	224 (37.3%)	187 (26.7%)
Toilet = pit latrine	364 (60.7%)	417 (59.6%)
Household owns at least one bicycle	400 (66.7%)	483 (69.0%)
Household owns a radio	475 (79.2%)	553 (79.0%)
Household owns television	51 (8.5%)	86 (12.3%)
Household owns at least one mobile phone	384 (64.0%)	536 (76.6%)
Household connected to electricity	13 (2.2%)	5 (0.7%)
Household uses Firewood for cooking fuel	501 (83.5%)	621 (88.7%)
Household owns > = 10 cows	21 (3.5%)	34 (4.9%)
Household walls made from brick/cement	135 (22.5%)	115 (16.4%)
Household walls made from clay/mud	458 (76.3%)	579 (82.7%)
Household floor made from cement	145 (24.2%)	146 (20.9%)
Household floor made of earth	454 (75.7%)	553 (79.0%)
Household head education		
Primary complete	407 (67.8%)	508 (72.6%)
Secondary or higher	168 (28.0%)	
Number of caregivers interviewed	628	717

* *Refers to households that own the structure/house in which they live in*.

### Fever treatment seeking behaviour pre- (2009) post- (2010) intervention

Table 2 shows the proportion of febrile children for whom any treatment was sought (including home-medication with ‘western medicines’ but not including prayers, or use of herbal medicines) and those for whom any treatment was sought within 48 hours across the two surveys. In both surveys most caregivers of febrile children reported seeking some form of treatment. However, there was a increase from 87.3% in 2009 to 91.8% in 2010, in the proportion of febrile children who sought any treatment (p = 0.02; Table 2). There was also an increase from 62.8% in 2009 to 79.4% in 2010 (p<0.001; Table 2) in caregivers who reported seeking formal treatment promptly (on the same day, or next day) for their febrile children. Sources of treatment for fever were similar between observation periods, but the proportion of fevers where first treatment actions included the formal public sector declined from 39.5% in 2009 to 22.3% in 2010 following the intervention (p = <0.001, Table 2). A decrease from 23.1% to 16.5% was also observed in the proportion of febrile children accessing treatment from the public sector within 48 hours (p = 0.04, Table 2) between the 2009 and 2010 surveys. There was little variation pre- and post-intervention in the proportions of caregivers first seeking treatment from the retail sector (29.4%, in 2009 and 31.4% in 2010 or from private clinics (3.0%) in 2009 and 4.0% in 2010 (Table 2). There were, however, changes in the proportion of childhood fevers managed with drugs available within the household: from 12.4% in 2009 to 31.1% in 2010 (p = <0.001, Table 2). Community health workers were rarely accessed as the first source of fever treatment in either 2009 or 2010.

**Table 2:** Caregivers first source of treatment seeking and antimalarials received for febrile children < 5 years of age.

	Pre-intervention (June-July 2009)	Post-intervention (July-August 2010)		
	n (%)	n (%)	Difference in proportions (95% CI)	P- value*****
**Fevers and source of treatment (First action)**				
Total Number of children <5 years of age	958	1,123		
Proportion of children< 5 years reporting fever in last 14 days	506 (52.8)	515 (45.9)	-6.9 (-11.1, -2.6)	0.006
Proportion of children< 5 years reporting fever in last 14 days but not febrile on day of survey	414 (81.8)	377 (73.2)	-8.6 (13.7, -3.5)	0.001
Number of children< 5 years reporting fever in last 14 days but not febrile on day of survey	N = 506	N = 515		
Proportion of all febrile children seeking any treatment (not including prayers)	442 (87.3)	473 (91.8)	4.5 (0.7, 8.2)	0.02
Proportion of all febrile children seeking any treatment (not including prayers) within 48 hours	318 (62.8)	409 (79.4)	16.6 (11.1, 22.1)	<0.001
Proportion febrile children accessing treatment from formal government of Kenya (GoK)/mission health facilities	200 (39.5)**	115 (22.3)**	-17.2 (-22.8,-11.6)	<0.001
Proportion febrile children accessing treatment from formal government of Kenya (GoK)/mission health facilities within 48 hours	117 (23.1)	85 (16.5)	-6.6 (-11.5, -1.7)	0.04
Proportion febrile children accessing treatment from formal private sector	14 (3.0)	20 (4.0)	1.0 (-1.2, 3.2)	0.43
Proportion of febrile children accessing treatment from formal retail sector	149 (29.4)	162 (31.4)	2.0 (-3.6, 7.6)	0.63
Proportion of febrile children accessing treatment from drugs available in household	63 (12.4)	160 (31.1)	18.7 (13.8, 23.6)	<0.001
Proportion of febrile children accessing treatment from community health workers (CHWs)	16 (3.2)	16 (3.1)	-0.1 (-2.2, 2.0)	1.00
Proportion of all febrile children not seeking any treatment	64 (12.7)	42 (8.2)	-4.5 (-8.2, -0.7)	0.02
**Drugs used to treat malaria**				
Proportion of febrile children accessing any anti-malarial drug in last 14 days	224 (44.3)	202 (39.2)	-5.1 (-11.1, 0.9)	0.12
Proportion of febrile children accessing AL at any time in last 14 days	157 (31.0)***	165 (32.0)****	1.0 (-4.7, 6.7)	0.76
Proportion of febrile children treated with quinine	33 (6.5)	27 (5.2)	-1.3 (-4.2, 1.6)	0.44
Proportion of febrile children treated with sulfadoxine-pyrimethamine (SP)	5 (1.0)	6 (1.3)	0.3 (-1.0, 1.6)	0.76
Proportion of febrile children treated with amodiaquine	38 (7.5)*****	9 (1.7)	-5.8 (-8.3, -3.2)	<0.001
Proportion of febrile children treated with chloroquine	3 (0.6)	1 (0.2)	-0.4 (-1.2, 0.4)	0.30
Proportion of febrile children treated with dihydroartemisinin + piperaquine	1 (0.2)	0	-0.2 (-0.6, 0.2)	-

*Proportions are cluster adjusted

**Range of proportion across clusters in 2009 (0–61.*5%%); 2010 (0–83*.*3%)*

***Includes 10 febrile children treated with quinine injections and AL;

****Includes 7 febrile children treated with quinine injections and AL

*****Includes one febrile child treated with amodiaquine and quinine injection

*****P-value from chi-squared test adjusted for clustering

### Antimalarial treatments for febrile-children pre-post intervention

Of the 506 febrile children in the 2009 survey, the proportion accessing any antimalarial drug in the last 14 days was 44.3%, similar to the 39.2% of the 515 febrile children who were reported to have accessed antimalarial drugs during the 2010 survey (p = 0.13, Table 2). In addition, there were no changes in the proportion of febrile children accessing AL at any time in the last 14 days between the two observation periods, 31.0% (2009) versus 32.0% (2010) (Table 2). There was, however, a decline in the proportion of febrile children accessing amodiaquine from 7.5% in 2009 to 1.7% in 2010 (p = <0.001, Table 2). Six percent (33) of febrile children in 2009 and 5.2% (27) in 2010 received quinine while a few of the febrile children received ineffective and/or non-recommended monotherapies ineffective and/or non-recommended monotherapies (Table 2). Across both surveys, only one child received dihydroartemisinin-piperaquine, launched as the recommended second-line antimalarial in August 2010 (Table 2).

### AL treatment, timing and adherence

For the primary indicator, *‘the proportion of children under five years of age with fever in the last 14 days accessing AL within 48 hours of fever onset’* there was a 5.1 percentage point increase from baseline 18.4% to 23.5% post-intervention (p = 0.06, Table 3). However, there were no increases in the proportion of febrile children accessing AL from a public health facility within 48 hours of the onset of fever (Table 3). There was an increase in the numbers of febrile children treated using AL available from within the household from 1.6% to 5.4% between 2009 and 2010 (Table 3: 95% CI: 1.6, 6.0) but, since no data were collected on the original source of the drugs which were used ‘from within the household’, it is not possible to identify where these drugs originally came from. Information on the source of drugs from outside the household was collected and, although there were no increases in the proportion of febrile children accessing the retail sector as the first source of fever treatment, there was a significant increase in the proportion of febrile children whose fever treatment included AL obtained from the retail sector. The proportion of febrile children who received treatment with AL that had been sourced from the retail sector rose from 1.4% in 2009 to 4.0% in 2010) (p = 0.01, Table 3), suggesting that AL was becoming more available in this sector and that caregivers were more aware of AL and asked for it from the retail shops.

**Table 3:** Comparison of AL treatment, timing and adherence between pre- (2009) and post-intervention (2010). All proportions are cluster adjusted

	Pre-intervention (June-July 2009)	Post-intervention (July-August 2010)		
	n/N (%)	n/N (%)	Difference in proportion (95% CI)	P- value**
**Source of AL treatment**				
Public sector	137/506 (27.1)	108/515 (21.0)	-6.1 (-11.3, -0.9)	0.07
Formal private sector	5/506 (1.0)	10/515 (1.9)	0.9 (-0.6, 2.4)	0.30
Retail commercial sector	7/506 (1.4)	19/515 (4.0)	2.6 (0.6, 4.6)	0.01
Drugs available in household	8/506 (1.6)	28/515 (5.4)	3.8 (1.6, 6.0)	<0.001
**AL treatments where treatment started ≥ 4 days ago**	147/157 (93.6)	152/165 (92.1)	-1.5 (-7.1, 1.4)	0.60
Of all AL treatments where treatment started ≥ 4 days ago number who completed dose	101/147 (68.7)	127/152 (83.5)	14.8 (5.3, 24.3)	0.01
Timing and adherence of AL treatment				
Proportion of febrile children accessing AL within 48 hours	93/506 (18.4)*	121/515 (23.5)*	5.1 (0.1, 10.0)	0.06
Proportion of fevers children AL within 48 hours from a public health facility	80/506 (15.8)	70/515 (13.6)**	-2.2 (-6.5, 2.1)	0.39
Of all AL treatments within 48 hours and where treatment started > = 4 days ago number who completed dose	56/93 (60.2)	78/121 (64.5)	4.3 (-8.8, 17.4)	0.58
Of all AL treatments within 48 hours from a public health facility and where treatment started ≥ 4 days ago number who completed dose	54/80 (67.5)	57/70 (81.4)	13.9 (0.2, 27.6)	0.05
Proportion of caregivers who sourced AL from public sector who had dose explained	128/137 (93.4)	101/108 (93.5)	0.1 (-6.2, 6.3)	0.98

*Range of proportion across clusters in 2009 (0–33.9%); 2010 (0–52.9%)

**P-value from chi-squared test adjusted for clustering

In 2009, 147 (93.6%) and in 2010, 152 (92.1%) of all AL treatments were started four or more days prior to the survey day (Table 3). Of these, there was an increase in the proportion who reported adhering to the complete course of AL treatment, from 68.7% in 2009 to 83.5% in 2010 (p = 0.01, Table 3). Initiating treatment promptly was not associated with the likelihood of dosage completion. Of all AL treatments that had been started within 48 hours of fever onset and where the initial dose had been taken four or more days prior to the survey there was no significant difference in the proportion of febrile children who were reported to have received a complete course of treatment: 60.2% in 2009 and 64.5% in 2010 (p = 0.58, Table 3). It is possible that the lack of change in adherence rates with increased promptness of treatment was influenced by the higher rate of treatments sourced from within the home in 2010 since these treatments were associated with high non-completion rates. Of the 28 febrile children in the 2010 survey who were reported to have been given AL that was obtained from within the household only nine (32.1%) reported that a complete course of drugs had been available at the start of the treatment and that the full dose had been taken. When the analysis was restricted to those where treatment was sought promptly at a public health facility, there was a significant increase in adherence from 67.5% in 2009 to 81.4% in 2010 (p = 0.05, Table 3).

### Predictors of fever treatment seeking behaviour and prompt access to artemether-lumefantrine

In 2009, none of the variables were associated with prompt access to AL (Table 4). In 2010, univariable logistic regression showed that children aged two years and above were four times (p = 0.03, Table 5) more likely to access prompt fever treatment with AL compared with children aged one year and below. Febrile children in less poor (quintile 4) households were three times more likely to access AL within 48 hours of fever onset compared to those in poorer quintiles (p = 0.05, Table 6). In the final regression model in 2010 only child’s age (p = 0.03, Table 6) remained associated with prompt access to AL.

**Table 4:** Univariable and multivariable logistic regression analysis of factors predicting prompt (within 48 hours) treatment of fever with AL among children < 5 years of age in 2009.

			Univariable		Multivariable	
Variable	No. of febrile children	N (%) febrile children with outcome	OR (95% CI)	P value	OR (95% CI)	P value
**Child age**						
<1 year^a^	90	18 (20.0)	1.0		1.0	
1 year	118	17 (14.4)	0.5 (0.1, 1.6)	0.23	0.3 (0.1, 1.3)	0.11
≥ 2 years	298	58 (19.5)	0.9 (0.3, 2.4)	0.83	0.8 (0.2, 2.5)	0.71
**Child gender**						
Male^a^	246	39 (15.8)	1.0		1.0	
Female	260	54 (20.8)	2.0 (0.9, 4.4)	0.10	2.5 (1.0, 6.5)	0.06
**Caregiver level of education**						
No education^a^	22	2 (9.1)	1.0		1.0	
Complete primary school	383	64 (16.7)	1.3 (0.2, 9.9)	0.81	2.2 (0.1, 32.7)	0.58
Complete secondary or higher	101	27 (6.7)	5.2 (0.6, 47.1)	0.14	5.3 (0.3, 94.2)	0.26
**Caregiver age**						
<20 years^a^	42	6 (14.3)	1.0		1.0	
20–30 years	303	57 (18.8)	1.7 (0.4, 8.2)	0.47	1.2 (0.2, 8.1)	0.83
31–40 years	109	23 (21.1)	1.8 (0.3, 9.7)	0.49	1.0 (0.1, 7.7)	0.98
41–50 years	41	6 (14.6)	1.0 (0.1, 7.7)	0.99	0.5 (0.04, 6.7)	0.62
>50 years	11	1 (9.1)	0.5 (0.1, 14.1)	0.66	0.2 (0.002, 13.4)	0.43
**Number of children under five years in household**						
One child^a^	222	43 (19.4)	1.0		1.0	
≥ two children	284	50 (17.6)	0.8 (0.4, 1.8)	0.67	0.6 (0.2, 1.7)	0.34
**Socio-economic category of household**						
Quintile 1 (most poor)^a^	112	17 (15.2)	1.0		1.0	
Quintile 2 (very poor)	86	21 (24.4)	3.4 (0.8, 13.6)	0.08	4.0 (0.8, 19.6)	0.08
Quintile 3 (poor)	106	24 (22.6)	2.9 (0.8, 10.9)	0.11	4.0 (0.9, 18.5)	0.08
Quintile 4 (less poor)	99	16 (16.2)	1.3 (0.3, 4.9)	0.70	1.0 (0.2, 5.1)	0.94
Quintile 5 (least poor)	103	15 (14.6)	1.1 (0.3, 4.0)	0.91	0.7 (0.1, 3.5)	0.68
**Distance to nearest public health facility**						
**<1 km** ^**a**^	72	15 (20.8)	1.0		1.0	
**1-<2km**	96	15 (15.6)	0.6 (0.1, 2.6)	0.53	0.6 (0.1, 3.1)	0.54
**2-<3km**	144	26 (18.1)	0.8 (0.2, 3.0)	0.78	0.7 (0.1, 3.1)	0.63
**3-<4km**	116	25 (21.5)	1.2 (0.3, 4.4)	0.81	1.4 (0.3, 6.9)	0.65
**4-<5km**	63	9 (14.3)	0.4 (0.1, 2.3)	0.34	0.4 (0.1, 2.7)	0.35
**≥5km**	15	3 (20.0)	1.3 (0.1, 15.6)	0.84	2.8 (0.1, 54.4)	0.50

^a^ Reference group

**Table 5:** Univariable and multivariable logistic regression analysis of factors predicting prompt (within 48 hours) treatment of fever with AL among children < 5 years of age in 2010.

			Univariable		Multivariable	
Variable	No. of febrile children	N (%) febrile children with outcome	OR (95% CI)	p-value	OR (95% CI)	p-value
**Child age**						
<1 year^a^	90	15 (17.0)	1.0		1.0	
1 year	105	19 (18.5)	1.4 (0.3, 5.7)	0.63	1.5 (0.3, 7.0)	0.56
≥ 2 years	320	87 (27.2)	4.5 (1.2, 17.4)	0.03	5.5 (1.3, 22.9)	0.02
**Child gender**						
Male^a^	257	61 (23.7)	1.0		1.0	
Female	258	60 (23.3)	0.9 (0.4, 1.9)	0.83	0.8 (0.3, 1.9)	0.65
**Caregiver level of education**						
No education^a^	10	0 (-)				
Complete primary school	425	95 (22.3)	1.0		1.0	
Complete secondary or higher	80	26 (32.5)	2.4 (0.8, 6.8)	0.10	2.2 (0.6, 7.9)	0.20
**Caregiver age**						
<20 years^a^	43	7 (16.3)	1.0		1.0	
20–30 years	307	80 (26.1)	2.9 (0.6, 13.8)	0.18	2.2 (0.3, 13.6)	0.40
31–40 years	111	21 (18.9)	1.4 (0.3, 7.5)	0.67	0.7 (0.1, 5.5)	0.79
41–50 years	41	9 (21.9)	1.9 (0.3, 13.7)	0.53	1.5 (0.1, 16.1)	0.72
>50 years	13	4 (31.0)	4.7 (0.3, 71.8)	0.26	4.3 (0.1, 115.6)	0.39
**Number of children under five years in household**						
One child^a^	210	53 (25.2)	1.0		1.0	
≥ two children	305	68 (22.3)	0.8 (0.4, 1.8)	0.63	1.0 (0.4, 2.6)	0.98
**Socio-economic category of household**						
Quintile 1 (most poor)^a^	117	23 (20.0)	1.0		1.0	
Quintile 2 (very poor)	105	13 (12.4)	0.4 (0.1, 1.7)	0.24	0.3 (0.1, 1.5)	0.15
Quintile 3 (poor)	117	29 (25.0)	2.0 (0.6, 6.7)	0.25	1.5 (0.4, 5.9)	0.56
Quintile 4 (less poor)	98	32 (32.6)	3.5 (1.0, 12.3)	0.05	2.7 (0.6, 11.9)	0.19
Quintile 5 (least poor)	78	24 (31.0)	3.2 (0.8, 12.3)	0.08	1.8 (0.4, 8.6)	0.43
**Distance to nearest public health facility**						
<1 km^a^	37	11 (29.7)	1.0		1.0	
1-<2km	217	48 (22.1)	0.5 (0.1, 2.2)	0.36	0.3 (0.1, 1.9)	0.20
2-<3km	123	31 (25.2)	0.7 (0.1, 3.2)	0.61	0.4 (0.1, 2.9)	0.40
3-<4km	68	16 (23.5)	0.4 (0.1, 2.4)	0.31	0.2 (0.02, 1.9)	0.16
4-<5km	54	13 (24.1)	0.6 (0.1, 3.6)	0.59	0.3 (0.03, 2.6)	0.27
≥5km	16	2 (12.5)	0.2 (0.1, 3.2)	0.23	0.1 (0.002, 1.7)	0.10

^a^ Reference group

**Table 6:** Final multivariable regression of factors predicting prompt (within 48 hours) treatment of fever with AL among children < 5 years of age in 2010*- (factors with p-value <0*.*05)*.

Variable	OR (95% CI)	p-value
**Child age**		
<1 year^a^	1.0	
1 year	1.4 (0.3, 6.0)	0.63
≥ 2 years	4.6 (1.2, 18.2)	0.03

^a^ Reference group

### AL availability at government and mission health facilities

Over 70% of health facilities had any AL blister packs in stock across the two survey periods. Between April and May 2010, less than half of health facilities had AL paediatric packs (Table 7). Over 80% of health facilities had the adult AL blister packs (AL 24 tablets) in stock during the audit period. At no point during the 10 months did all health facilities have all AL blister packs in stock (Table 7). Less than half of health facilities had all AL blister packs in stock two months prior to the pre- and post-intervention surveys. Furthermore, only 41.5% and 19.5% of health facilities had all AL blister packs in April and May 2010. The study was predicated on measuring access and use of AL from the public sector. Prior to each household sample survey efforts were made to ensure adequate stocks of pediatric pack sizes were available at least four weeks before the survey began and during the survey period to ensure universal availability of AL at public health facilities. Two months prior to the post-intervention survey, less than half of all facilities had AL paediatric packs in stock; however, we ensured that AL was delivered to all health facilities in Bondo two weeks prior to the survey.

**Table 7:** AL availability (all AL pack sizes) assessed by telephone interviews with health facilities (TI) or directs observation through physical audit (PA) at 33 government facilities in 2009 and 41 government and mission facilities in 2010 in Bondo district. Note: Emergency supplies sent on 22^nd^ July 2010 to facilitate survey. AL 18 tablets (25-<35kgs) blister packs were not available for this emergency supply. In June and July 2010, six and twelve blister packs were Coartem Dispersible;

	Number of health facilities	Any AL blister packs in stock	AL 6 tablets blister pack in stock	AL 12 tablets blister pack in stock	AL 18 tablets blister pack in stock	AL 24 tablets blister pack in stock	All AL blister packs in stock
	N	n (%)	n (%)	n (%)	n (%)	n (%)	n (%)
**January 2009 (TI)**	33	25 (75.8)	16 (48.5)	22 (66.7)	15 (45.4)	19 (57.6)	10 (30.3)
**May 2009 (TI)**	33	33 (100)	14 (42.4)	33 (100)	15 (45.4)	33 (100)	14 (42.4)
**June 2009 (PA)**	33	33 (100)	33 (100)	33 (100)	31 (93.9)	33 (100)	31 (93.9)
**October 2009 (TI)**	33	31 (93.9)	31 (93.9)	31 (93.9)	31 (93.9)	30 (90.9)	30 (90.9)
**January 2010 (TI)** *	41	40 (97.6)	27 (65.8)	37 (90.2)	37 (90.2)	37 (90.2)	24 (58.5)
**April 2010 (TI)** *	41	38 (93.0)	19 (46.3)	25 (61.0)	29 (70.7)	37 (90.2)	17 (41.5)
**May 2010 (TI)** *	41	32(78.0)	13(31.7)	18(43.9)	17(41.5)	33(80.5)	8(19.5)
**June 2010 (TI)** *	41	37 (95.0)	35 (89.7)	35 (89.7)	34 (87.2)	36 (92.3)	33 (84.6)
**July 2010 (PA)** *	41	41 (100)	41 (100)	41 (100)	18 (43.9)	41 (100)	18 (43.9)
**August 2010 (PA)** *	41	39 (95.1)	39 (95.1)	38 (93.0)	10 (24.4)	37 (90.2)	10 (24.4)

*Includes three new government health facilities established and commissioned in January 2010 through the Constituency Development Fund (CDF) initiative and five mission health facilities

## Discussion

The *haraka upesi* behaviour change communication intervention to improve prompt and effective treatment for children under five with a febrile illness targeted three steps on the fever treatment seeking pathway: 1) prompt fever treatment, 2) fever treatment at a public health facility and 3) adherence to the AL treatment regimen.

Overall, there were improvements in two key steps: prompt fever treatment and prompt access to AL, with a 5.1 percentage point increase observed in the primary outcome. In Bondo district, the majority of caregivers of children under five years of age with a fever sought treatment for their child both before the implementation of the intervention in 2009 (87.3%) as well as post intervention in 2010 (91.8%). There was a significant increase in prompt (within 48 hours) treatment seeking between 2009 and 2010 but this did not translate into increased access to anti-malarial treatment, with only approximately 40% of fevers (44.3% in 2009 and 39.2% in 2010) receiving any anti-malarial. However, the proportion of febrile children accessing AL (32%) and accessing AL promptly (23%) in the post intervention period appears to be relatively high when compared to reports from earlier surveys on treatment seeking behaviour undertaken in Bondo district and in Kenya. The study by Gitonga and colleagues conducted in Bondo district between 2006 and 2007 found that only 11% of caregivers with a febrile child under 5 had accessed AL within 48 hours of fever onset [7]. It is possible that these differences are related to the short time that had elapsed between the implementation of the AL policy and the conduct of these surveys.

Data from the most recent World Malaria Report shows that the proportion of febrile children accessing antimalarials in SSA ranges from 5.7% to 70% [20] and in most countries fewer than 20% of febrile children access ACTs promptly [21]. Despite interventions aimed at increasing prompt AL treatment in Bondo considerable efforts are required, as elsewhere in Africa, to reach the the RBM target of 80%.

In Bondo, the proportion of febrile children whose caregivers reported using the government sector as the first source for fever treatment declined despite the focus of the communication initiative being around prompt fever treatment at a public health facility. A recent multicountry study found that only 13.4% of febrile children in Benin, 16% in Uganda and the Democratic Republic of Congo (DRC), and 17.6% in Nigeria had been taken for treatment in a public health facility [22]. Low use of government health facilities is not universal, however, and a study undertaken in Zambia reported almost half (49.8%) of febrile children accessed a public health facility for fever treatment [22], while public health facility utilization of ≥50% for fever treatment has been reported in Tanzania, Namibia, Mozambique, Djibouti, Sudan and Liberia [23]. A study undertaken in Tanzania following the roll out an intervention similar to *haraka upesi* reported that more half (58%) of febrile children accessed a health facility as the first source for fever treatment, however, general health facility attendance among children was already high (76%) at baseline [24]. In general, in Kenya the reported use of the government sector as the first source of treatment for febrile children ranges from 29.3% to 33.6% [23,25], lower than in Tanzania. In the current study undertaken in Bondo district Kenya, at baseline in 2009, 39.5% of the caregivers reported that their first treatment action for a febrile child was to take them to a government health facility and this dropped to 22.3% in 2010.

A key factor that has been found to influence treatment seeking behaviour for a febrile illness is the availability of drugs [24, 25–29]. Understandably, most caregivers will seek treatment from sources where they are sure of receiving treatment with a drug. Nationwide stock outs of AL at public health facilities were reported at the onset of this study between 2008 and 2009 and AL availability was variable throughout the entire study period. The political situation in Kenya at the onset of the study and delays in AL procurement affected AL supply in Bondo district. Supply side problems in the public sector have been widely document as a key factor affecting its use [27, 30–32]. It therefore seems likely that fluctuations in AL supply might have led to a lack of patient confidence in our study population in the public sector’s ability to provide treatment for fever which contributed to the drop off in use of government health facilities as the first source of treatment that was observed in the post-intervention period.

A more surprising observation was the increase between 2009 and 2010 in the number of reports of first treatments of a febrile child being undertaken with drugs sourced from within the household. It is possible that, in response to the fluctuations in AL supply mentioned previously, caregivers had become inclined to hoard AL packs in the household leading to the observed increase in the proportion of home treatments (and home treatment with AL) in the post-intervention survey. Of concern is the fact that only one third of the AL treatments sourced from within the home were reported to have started with complete blisters packs while two thirds of AL drugs obtained within the households were incomplete packs. The presence of incomplete AL packs in the household and their use in subsequent febrile episodes has implications for correct dosing as it suggests that febrile children are receiving inadequate doses. AL is taken over a three-day period and has a complex dosage scheduled. Several studies have reported varying adherence rates for ACTs of between 38.7% and 93% [33–45] and poor adherence to AL has been suggested as a major constraint to its effectiveness [37, 44, 46]. In the current study reported adherence to AL increased from around two thirds (67%) to over 80% among caregivers who sourced their treatment from the public health facility but this increase was not reflected in overall adherence rates, perhaps due to the decline in the use of the government sector.

The evaluation design included a post-intervention comparison to measure changes in the fever treatment seeking indicators. We made adjustments for multiple comparisons using the Bonferroni procedure [0.001 (0.05/34)]. However, the analyses presented in this study, do not allow the attribution of any of the observed changes in the primary and secondary indicators to reported exposure to the intervention. That is, the analysis provides an adequacy inference with no attempt to attribute the observed changes to the *haraka upesi* intervention.

The Intervention reported in this study was a complex intervention implemented under routine operational conditions. Such interventions present particular problems for evaluators, as they contain several interacting components, they often involve a number of different behaviours and they tend targets different levels within a system (for example household and health facility). In addition to the complexity of the intervention, a key strategy in the BCC campaign was the use of mass media (radio). Where mass media are employed it is difficult to identify an appropriate control group that is not exposed to the intervention. Under these conditions it was not possible to employ a probability evaluation. In this evaluation we undertook a process evaluation to explore the way in which the intervention had been implemented and outcome data in this study was collected using pre- and post-cross-sectional community surveys to assess changes in caregiver treatment seeking behaviour. Overall, the evaluation cannot completely isolate the effect of the *haraka upesi* intervention In this evaluation we undertook a process evaluation to explore the way in which the intervention had been implemented and outcome data in this study was collected using pre- and post-cross-sectional from those of other concurrent processes or activities. Furthermore, the use of pre- and post-intervention evaluation design does not account for the changes that occurred between the two time points that were not related to the intervention. The time interval between intervention implementation and evaluation has been has also been highlighted as a methodological challenge in the evaluation of BCC (www.cpc.unc.edu/measure/prh/rhindictators/crosscutting/bcc accessed 25/07/2013).

### Conclusion

Overall the data suggest that, during the pre- and post-intervention period the biggest barrier to effective treatment for febrile children under five in Bondo district was the infrequent use of the government health facilities as the first step in treatment seeking. There was evidence of a significant shift in treatment seeking behaviour away from the government sector (the intervention target delivery point) and the likely reasons for the shift in source of treatment relates to the implementation context mentioned above (drug stock-outs) which lay outside of the control of the intervention. These data demonstrate the importance of understanding the implementation context when analysing and interpreting intervention outcomes. The context may be the driver of how and whether the intervention works. They also demonstrate that in contexts where treatment for febrile illnesses is available from more than one sector, interventions that target only one sector will struggle to produce population level improvements in prompt and effective treatment. To make a real impact upon effective case management of febrile children at the national level, cross-sectoral interventions are needed. These are interventions across the potential sources of fever treatment and include public and mission, private and retail sectors.
